# Humoral immunity to SARS-CoV-2 in kidney transplant recipients and dialysis patients: IgA and IgG patterns unraveled after SARS-CoV-2 infection and vaccination

**DOI:** 10.1186/s12985-024-02410-1

**Published:** 2024-06-13

**Authors:** Caroline De Bouver, Jason Bouziotis, Veerle P. W. M. Wijtvliet, Kevin K. Ariën, Joachim Mariën, Leo Heyndrickx, Marie M. Couttenye, Hans J. W. de Fijter, Fabienne Mestrez, Serge Treille, Olivier Mat, Frederic Collart, Sabine D. Allard, Lies Vingerhoets, Pieter Moons, Daniel Abramowicz, Benedicte Y. De Winter, Lissa Pipeleers, Karl Martin Wissing, Kristien J. Ledeganck

**Affiliations:** 1 Laboratory of Experimental Medicine and Pediatrics and member of the Infla-Med Centre of Excellence, University of Antwerp, Antwerp, Belgium; 2 Department of Biomedical Sciences, University of Antwerp, Antwerp, Belgium; 3 Clinical Trial Center (CTC), CRC Antwerp, Antwerp University Hospital, Edegem, Belgium; 4 Present Address: Department of Nephrology and Hypertension, Antwerp University Hospital, Edegem, Belgium; 5 Virology Unit, Department of Biomedical Sciences, Institute of Tropical Medicine, Antwerp, Belgium; 6 Department of Nephrology-Dialysis, University Hospital (CHU) Ambroise Paré, Mons, Belgium; 7 Department of Nephrology, Centre Hospitalier Universitaire Charleroi, Charleroi, Belgium; 8 Department of Nephrology, Hospital Centre EpiCURA, Ath, Belgium; 9 Department of Nephrology, Hospital Universitaire Brugmann, Brussels, Belgium; 10 Department of Internal Medicine and Infectious Diseases, Vrije Universiteit Brussel, Universitair Ziekenhuis Brussel, Brussels, Belgium; 11 Department of Nephrology, AZ Turnhout, Turnhout, Belgium; 12 Biobank Antwerp, Antwerp University Hospital, Edegem, Belgium; 13 Department of Nephrology, Universitair Ziekenhuis Brussel, Vrije Universiteit Brussel, Brussels, Belgium

**Keywords:** COVID-19, Dialysis, Immune response, Kidney transplantation, SARS-CoV-2 vaccination

## Abstract

**Background:**

Infection with SARS-CoV-2 in high-risk groups such as kidney transplant and dialysis patients is shown to be associated with a more serious course of the disease. Four years after the start of the COVID-19 pandemic, crucial knowledge on the immune responses in these patient groups is still lacking. Therefore, this study aimed at investigating the humoral immune response after a SARS-CoV-2 infection compared to vaccination as well as the evolution of immunoglobulins over time.

**Methods:**

Kidney transplant recipients, patients on haemodialysis or on peritoneal dialysis and healthy controls were included in this longitudinal multicenter study. SARS-CoV-2 anti-RBD, anti-NP and anti-S1S2 immunoglobulin G (IgG) and A (IgA) as well as the neutralizing antibody capacity were measured.

**Results:**

Kidney transplant recipients had a significantly better humoral response to SARS-CoV-2 after infection (86.4%) than after a two-dose mRNA vaccination (55.8%) while seroconversion was comparable in patients on haemodialysis after infection (95.8%) versus vaccination (89.4%). In individuals without prior COVID-19, the IgG levels after vaccination were significantly lower in kidney transplant recipients when compared to all other groups. However, the IgA titres remained the highest in this patient group at each time point, both after infection and vaccination. A history COVID-19 was associated with higher antibody levels after double-dose vaccination in all patient categories and, while decreasing, titres remained high six months after double-dose vaccination.

**Conclusion:**

Kidney transplant recipients had a more robust humoral response to SARS-CoV-2 following infection compared to a two-dose mRNA vaccination, while patients on haemodialysis exhibited comparable seroconversion rates. Notably, individuals with prior COVID-19 exhibited higher IgG levels in response to vaccination. Hybrid immunity is thus the best possible defence against severe COVID-19 disease and seems also to hold up for these populations. Next, it is not clear whether the higher IgA levels in the kidney transplant recipients is beneficial for neutralizing SARS-CoV-2 or if it is a sign of disease severity.

**Supplementary Information:**

The online version contains supplementary material available at 10.1186/s12985-024-02410-1.

## Key learning points

**What was known**

- In high-risk groups such as kidney transplant patients and dialysis patients, the immune system does not function optimally due to several metabolic processes and lifelong use of immunosuppressants.

- Severe infection with SARS-CoV-2 in these high-risk groups is known to be associated with a more serious course of the disease.

- Severe disease after SARS-CoV-2 infection was rarely reported but all concerned KTRs who lacked immune responses after vaccination.

- Antibody levels decreased four weeks post-vaccination, which emphasizes the importance of booster vaccines.

- Kidney transplant recipients with prior COVID-19 exhibited higher anti-Spike IgG levels after a single dose of a SARS-CoV-2 mRNA-based vaccine.

**This study adds**

- Humoral immune response to SARS-CoV-2 in kidney transplant recipients was more robust following infection than vaccination.

- Next to kidney transplant recipients, patients on dialysis with prior COVID-19 exhibited higher anti-RBD and anti-S1S2 IgG levels as well, so hybrid immunity seems the best possible defence against severe COVID-19 disease in both populations.

- Kidney transplant recipients showed the highest IgA titres compared to any other group after double-dose vaccination and infection, along with the lowest IgG titres after vaccination.

**Potential impact**

- Our results strengthen the need to administer all the recommended vaccines to patients with end-stage renal disease, and to consider booster vaccination.

- Our findings make an important contribution to the COVID-19 research field by analysing both IgG and IgA serology and neutralizing antibody capacity after infection and vaccination, leading to a better understanding of the quality of the humoral immune response and its relationship with clinical data.

**Supplementary Information:**

The online version contains supplementary material available at 10.1186/s12985-024-02410-1.

## Introduction

The coronavirus disease 2019 (COVID-19) pandemic has raged for more than four years now. It is estimated that over 770 million individuals have been infected and that ∼seven million individuals have died (WHO) [1]. It is likely that the number of infections is even higher, as a large proportion of infected patients (85%) do not show serious signs of disease and have not been tested. Additionally, infection with severe acute respiratory syndrome coronavirus 2 (SARS-CoV-2) in high-risk groups such as kidney transplant recipients and patients who are on dialysis is known to be associated with a more serious course of the disease [2, 3]. In these patients, the immune system does not function optimally due to several metabolic processes and chronic use of immunosuppressants [4–6]. Both states of immunosuppression carry a greater risk of complications attributable to SARS-CoV-2 infection [6, 7] with mortality rates reported between 18.9% (Wuhan) and 52% (Lombardy) for haemodialysis patients and around 25% for kidney transplant patients [8–10]. Therefore, an effective vaccination strategy is important in these populations. An alternative to vaccination is the administration of (preventive) monoclonal antibodies in case of COVID-19 with increased risk of serious infection [11].

In the healthy population, it has already been shown that up to 90–95% of individuals develop a good immune response against SARS-CoV-2 after vaccination, however, they are less effective in high-risk groups such as solid organ transplant recipients [12]. It is likely that the immune response in kidney transplant patients and dialysis patients after vaccination will be weaker. Previous vaccination studies already showed less seroconversion after hepatitis B vaccination and a faster decay of the antibody titres in haemodialysis patients [13]. In kidney transplant recipients, lower seroconversion rates and antibody titres were reported after vaccination against influenza A/H1N1 and seasonal influenza [14–16]. Another recent study showed lower IgG and cellular immune responses in both haemodialysis and kidney transplant patients four weeks post-booster mRNA vaccination [17]. On the other hand, the antibody response in infected patients remains largely unknown and it remains uncertain whether the antibody response induced by infection protects against new infectious episodes. Around 93% of individuals develop IgM and 83% of individuals develop IgG against SARS-CoV-2, although we expect that these seroconversion rates are lower in high-risk groups [18]. The major targets of antibodies are the spike protein expressed by SARS‐CoV‐2 and its other structural protein the nucleocapsid protein (NP). The viral Spike protein engages with the angiotensin‐converting enzyme 2 (ACE2) receptor on the surface of the host cell, and the actual entry into the host cell is mediated by the receptor‐binding domain (RBD) of the S‐protein. Therefore, the spike protein is an important target for blocking virus entry, as occurs by antibodies induced through vaccination and in response to infection. [19, 20]. Secretory IgA might also play a major role in the protection against SARS-CoV-2, more specifically in the mucosae, and its contributions to humoral immunity and severe COVID-19 remains rather unexplored. Yet it has already been shown that serum IgA has a better neutralization activity than IgG against SARS-CoV-2 [21].

Severe disease after infection was rarely reported, but all involved KTRs who had no immune response after vaccination [22]. Breakthrough infections thus occur in KTRs when the vaccine was ineffective. Therefore, we wanted to investigate whether infection led to higher immunisation rates than vaccination, as a proof of concept that immunogenicity of vaccines needs further improvement. By including a large population of kidney transplant recipients (KTR), patients on haemodialysis (HD), patients on peritoneal dialysis (PD) and healthy controls into this longitudinal multicenter study, we aimed at investigating (i) whether there is a difference in humoral immune response (by the presence of IgG and IgA antibodies directed against SARS-CoV-2 antigens) after COVID-19 infection when compared to a two-dose mRNA SARS-CoV-2 vaccination, (ii) whether there is a difference in neutralizing capacity after COVID-19 infection when compared to a two-dose mRNA SARS-CoV-2 vaccination, (iii) the evolution of IgG, IgA and neutralizing antibody capacity over a six month time period, and (iv) which variables are associated with the antibody response.

## Materials and methods

### Patients and study protocol

Patients were recruited through three different studies: 1/ the SECRET study which included kidney transplant recipients, dialysis patients and healthy controls with a proven COVID-19 infection, 2/ the UPRAISE study which included kidney transplant recipients, dialysis patients and healthy controls after SARS-CoV-2 vaccination and 3/ the COVEMUZ study which included healthy volunteers after SARS-CoV-2 vaccination as explained in detail underneath.

SECRET (Study of the prevalence, sEroprevalence and seroConversion rate of the SARS-CoV-2 (COVID-19) viRus in kidney transplant patiEnts and haemodialysis patienTs) study: kidney transplant recipients (*n* = 22), haemodialysis patients (*n* = 24) and controls (*n* = 23) who were diagnosed with COVID-19 (Wuhan strain of SARS-CoV-2) were prospectively included into this study at the Antwerp University Hospital (UZA), CHU de Charleroi, Centre Hospitalier Universitaire Brugmann, Centre Hospitalier Universitaire Ambroise Paré and EpiCURA. Blood samples were collected at the moment of diagnosis, two months and five months thereafter. Blood tubes were centrifuged at 3 000 rpm during 10 min and serum was aliquoted and stored at -80°C at “Biobank Antwerp” (Antwerp, Belgium; ID: BE 71030031000)(Antwerp) [23] until further processing.

UPRAISE (Unraveling immune PRofiles After SARS-CoV-2 Immunization in dialySis and kidney transplant patiEnts) study: in this multicenter prospective longitudinal study consecutive kidney transplant recipients (*n* = 153) and patients on haemodialysis (*n* = 115) or peritoneal dialysis (*n* = 37) who received a SARS-CoV-2 vaccine between February and May 2021 were included at the UZA and Brussels University Hospital (UZ Brussel). Healthy controls were recruited through advertisement at the UZA website and the website of the University of Antwerp (*n* = 43). Patients under the age of 18 years and patient refusal were considered exclusion criteria. Patients were vaccinated with an mRNA SARS-CoV-2 vaccine according to the recommended scheme and blood samples were collected at five time points: before vaccination, 21 days after the first vaccine (and before the second vaccine), 56 days after the first vaccine (being 21 or 28 days after the second vaccine for BNT162b2 and mRNA-1273 respectively), six months and 12 months after the first vaccine. Serum samples (8ml) were centrifuged at 3 000 rpm for 10 min at room temperature and serum was aliquoted and stored at -80°C at “Biobank Antwerp” until further processing.

COVEMUZ study: 468 volunteers were recruited in this study through advertisement amongst employees of the UZBrussel between January 13th and February 5th 2021. [24] Of those, 130 were included in the data presented in this study based on age range (45-64y). Any adult employee of the UZ Brussel who provided signed informed consent to participate in the study was eligible for inclusion. Staff not active during the inclusion period were excluded. Serum samples were collected at three time points: baseline sample before SARS-CoV-2 vaccination, at day 28 after the second vaccine and six months after vaccination. Blood was centrifuged at 3 000 rpm during 10 min and stored at -20°C.

### Clinical data

Clinical data (such as age, sex, disease, transplantation and dialysis related data and treatment) were obtained from the patients’ electronic dossier in the respective hospital of inclusion. Data on age, sex and medical history were recorded via a questionnaire in healthy controls.

### Lab analyses

Immunoglobulin G (IgG) and A (IgA) antibodies against the receptor-binding domain (RBD), nucleocapsid protein (NP) and S1S2 of the SARS-CoV-2 spike protein were measured using an in-house Luminex assay [25]. These measurements were performed by staff unaware of the identity of the samples. In brief, our MIA is a high-throughput platform that allows the simultaneous detection of IgG antibodies against the different antigens from SARS-CoV-2. We diluted serum samples using a buffer (hypertonic Phosphate Buffer Saline buffer) to a final concentration of 1/300. The dilutions were mixed with 1.25 × 10^6^ paramagnetic MAGPLEX COOH-microsphere beads from Luminex Corporation (TX, USA) that were coupled with recombinant RBD, whole S protein and NP (BIOCONNECT, the Netherlands). After incubation of beads and diluted sera, we added biotin labeled anti-human IgG (1:125) and streptavidin-R-phycoerythrin (1:1000) conjugate. The beads were simultaneously read using a Luminex® Bio-Plex 100/200 analyser. We included an internal standard dilution with known international binding antibody units per ml (BAU/ml) to correct for interplate variation and allow comparison with different studies. We scored samples to be positive if antigens were higher than predefined cutoff levels (98% specificity and 95 sensitivity), which were estimated using a ROC analysis based on panel of validated negative of positive samples [25].

#### Virus Neutralization test

SARS-CoV-2 neutralising antibodies were quantified by incubating serial dilutions of heat-inactivated serum (1/50–1/25,600 in EMEM supplemented with 2mM L-glutamine, 100 U/ml–100 μg/ml of Penicillin–Streptomycin and 2% foetal bovine serum) during 1h (37 °C, 7% CO2) with 3xTCID100 of wild type (WT) lineage B virus (2019-nCoV-Italy-INMI1, reference 008V-03893). One hundred μl of sample-virus mixtures and virus/cell controls were added to 100 μl of Vero cells (18 000 cells/well) in a 96-well plate and incubated for five days (37 °C, 7% CO2). The CPE caused by viral growth was scored microscopically. The Reed–Muench method was used to calculate the nAb titre that reduced the number of infected wells by 50% (NT50), which was used as a proxy for the nAb concentration in the sample [25]. In accordance with WHO guidance, an internal reference standard is included in each nAb assay run. This internal standard was calibrated against the International Standard 21/234 (NIBSC) and NT50 values were recalculated to IU/ml for each variant [26]. A neutralization titre lower than 50, is considered to be negative.

### Definitions

#### Prior COVID-19 infection

patients with a PCR-proven history of SARS-CoV-2 infection and patients who tested positive for anti-RBD IgG antibodies (> 1) on the day of vaccination without a PCR-proven history of SARS-CoV-2 infection, as a surrogate of previous SARS-CoV-2 infection, were defined as prior COVID-19 infection.

#### Seroconversion

Response to first and second vaccination was defined as anti-RBD IgG positivity (signal-to-noise ratio > 1) at 16 to 21 days after the first dose, and 21 to 35 days after the second dose, respectively.

### Statistical analysis

We compared the frequencies of patients developing IgG and IgA antibodies and neutralizing capacity against SARS-CoV-2 between infected and vaccinated patients with Pearson’s Chi-squared test or Fisher’s exact test, depending on the expected frequencies. We compared the IgG and IgA levels between infected and vaccinated patients, and the IgG levels and neutralizing capacity between patients without and with prior COVID-19 exposure, with the Mann–Whitney-Wilcoxon test. We analyzed the correlation between IgG levels and neutralizing capacity, and between the time to blood sample and IgA and IgG levels, with Spearman’s correlation coefficient. We compared the IgG levels and neutralizing capacity between day 56 and month six with Wilcoxon signed-rank test. We analyzed the probability of antibody response with binomial logistic regression according to each patient characteristic separately (patient type, sex, age, comorbidities, years since last KTx, total number of KTx, years on dialysis, evidence of prior COVID-19, vaccine type and immunosuppressive categories), then built a multivariable model with the explanatory variables that were statistically significantly associated with the outcome, and reported the odds ratios with 95% confidence intervals. We analyzed the IgG level and neutralizing capacity with linear regression following the same procedure and reported mean differences with 95% confidence intervals. The p-values from pairwise comparisons were adjusted with Bonferroni’s correction. All tests were two-sided, and the significance level was set at 0.05. The analyses were performed with Stata/IC 15.1.

## Results

1. Immune response after SARS-CoV-2 infection vs. 2-dose SARS-CoV-2 mRNA vaccination in kidney transplant recipients (KTR), haemodialysis patients (HD) and controls.
  - Patients

- In total, 464 patients were included in the study of whom 69 were excluded for the following reasons: 15 HD patients with a history of kidney transplantation, 23 control patients in the vaccinated group with missing data on prior infection and 31 patients with overall missing data. 395 patients were included in the final analysis (151 kidney transplant recipients, 109 patients on haemodialysis and 135 controls). Baseline characteristics of the study population are shown in Supplemental Table 1.
  b. Development of IgG antibodies after SARS-CoV-2 infection vs. 2-dose SARS-CoV-2 mRNA vaccination

- The proportion of patients who developed IgG RBD and IgG S1S2 antibodies titres against SARS-CoV-2 were higher after infection than after vaccination in KTR (Table 1). In dialysis patients, the proportion of patients who developed IgG antibodies after vaccination was comparable to the infected group (*p* = 0.45). All control patients developed IgG antibodies after vaccination and after infection. Frequencies of IgG antibodies against nucleocapsid protein (NP) were not statistically significantly different between the patients (*p* = 0.31). Neutralizing capacity was significantly higher after vaccination in the control group (*p* = 0.001), but similar after infection or vaccination in all other groups. Within the infected group, the seroconversion rate did not differ between KTR, HD and controls. However, within the vaccinated group, the IgG anti-RBD and anti-S1S2 seroconversion rate was significantly lower in the KTR compared to HD and controls (both *p* < 0.001) and lower in HD compared to controls (*p* < 0.001).
- The antibody titres (both for RBD and S1S2) in responders were significantly higher after infection than after vaccination in the KTR group (Table 2). RBD antibodies were comparable between vaccination and infection in the HD group while S1S2 antibodies were significantly higher after infection. The control group, on the contrary, had higher Ab titres after vaccination versus after infection, both for RBD and S1S2. Within the vaccinated group, the titres were the lowest in KTR patients when compared to controls (p_adj_ < 0.001 for both RBD and S1S2) and HD (*p*_adj_ = 0.01 for RBD and *p*_adj_ = 0.001 for S1S2) while they were comparable between the three groups after infection (*p* = 0.73 for RBD and *p* = 0.11 for S1S2). In the infected group, anti-NP titres were significantly lower in KTR when compared to both other groups (*p* = 0.06).
- We also investigated if antibody titres could serve as a proxy for neutralizing capacity. Hitherto, the antibody titres were correlated to the neutralizing capacity in responders (details shown in Supplemental Table 2). Anti-RBD and anti-S1S2 antibodies did correlate well with the neutralizing capacity in the control group, both after vaccination (rs = 0.22 and 0.67 respectively) and infection (rs = 0.72 and 0.51 respectively). In HD, anti-RBD and anti-S1S2 Ab titres only correlated to neutralizing capacity after infection (rs = 0.64 and 0.47 respectively) but not after vaccination. In KTR, anti-RBD Ab did not correlate to neutralizing capacity and anti-S1S2 showed no correlation to neutralizing capacity after infection, while it strongly correlated after vaccination (rs = 0.78). As expected, there was no correlation between the neutralizing capacity and anti-NP IgG (data not shown). The lack of correlation between neutralizing capacity and IgG titres in some subgroups might be explained by low numbers of patients in the separate analyses, therefore, we performed a multiple linear regression of the neutralizing capacity including all patients (IgG responders with neutralizing capacity, *n *= 206). As shown in supplemental Table 3, neutralizing capacity was about 7% higher per 1-unit-increase in anti-RBD IgG titre and 36% lower in vaccinated people than in infected people but not different between patient types. Neutralizing capacity was about 9% higher per 1-unit-increase in anti-S1S2 IgG titre and 32% lower in vaccinated people than in infected people but not significantly different between patient types.
  c. Development of IgA antibodies after SARS-CoV-2 infection vs. 2-dose SARS-CoV-2 mRNA vaccination.

- IgA seroconversion happened significantly more after infection when compared to vaccination in all three groups both for anti-RBD as well as anti-S1S2 antibodies, with seroconversion in 73.9–80.9% of the patients after infection compared to 21.2–44.7% of the patients after vaccination for anti-RBD IgA (data shown in Table 3).
- Among responders, the IgA titres against S1S2 were significantly higher in the infected group compared to the vaccinated group as presented in Table 4. IgA anti-RBD titres were significantly higher in kidney transplant recipients and controls after infection versus after vaccination while in HD, the titres were comparable in both groups. IgA Ab titres did not correlate with the neutralization capacity (data not shown).
  d. Relation between timepoint of infection or first vaccine to determination of antibody titres

Table showing the proportion of patients who developed IgG antibodies against RBD, the S1S2 domain of SARS-CoV-2, NP and neutralizing capacity. Numbers highlighted in bold indicate statistical significance. *KTR* Kidney transplant recipients, *HD* hemodialysis, *RBD* receptor binding domain, *NP* nucleocapsid protein. *no variation

**Table 1:** Proportion of patients developing IgG antibodies and neutralizing capacity against SARS-CoV-2 after infection and vaccination

Infected			Vaccinated		
	*N*	*N* (%)	*N*	*N* (%)	*p*-value
**IgG antibodies against RBD**					
Overall	69	65 (94.2)	326	260 (79.7)	**0.004**
KTR	22	19 (86.4)	129	72 (55.8)	**0.007**
HD	24	23 (95.8)	85	76 (89.4)	0.45
Control	23	23 (100.0)	112	112 (100.0)	*
**IgG antibodies against S1S2**					
Overall	69	65 (94.2)	326	248 (76.1)	**0.001**
KTR	22	19 (86.4)	129	60 (46.5)	**0.001**
HD	24	23 (95.8)	85	76 (89.4)	0.45
Control	23	23 (100.0)	112	112 (100.0)	-
**IgG antibodies against NP**					
Overall	69	66 (95.7)		NA	
KTR	22	20 (90.9)		NA	
HD	24	23 (95.8)		NA	
Control	23	23 (100.0)		NA	
**Neutralizing capacity**					
Overall	69	62 (89.9)	152	145 (95.4)	0.14
KTR	22	20 (90.9)	21	17 (80.9)	0.41
HD	24	23 (95.8)	24	21 (87.5)	0.61
Control	23	19 (82.6)	107	107 (100.0)	**0.001**

Table showing the proportion of patients who developed IgG antibodies against RBD, the S1S2 domain of SARS-CoV-2, NP and neutralizing capacity. Numbers highlighted in bold indicate statistical significance. *KTR* Kidney transplant recipients; *HD* hemodialysis; *RBD* receptor binding domain, *NP* nucleocapsid protein. *no variation

**Table 2:** IgG antibody titres against SARS-CoV-2 in responders after infection and vaccination

	Infected		Vaccinated		
	*N*	Median [IQR]	*n*	Median [IQR]	*p*-value
**IgG antibodies against RBD**					
Overall	65	18.23 [9.38; 20.05]	260	19.33 [16.05; 20.90]	**0.02**
KTR	19	18.28 [12.97; 20.13]	72	10.85 [3.07; 18.72]	**0.02**
HD	23	16.82 [8.14; 20.46]	76	17.07 [15.68; 19.23]	0.77
Control	23	16.29 [9.30; 19.62]	115	19.93 [19.54; 22.44]	**< 0.001**
**IgG antibodies against S1S2**					
Overall	65	16.96 [10.32; 22.29]	248	16.64 [8.40; 21.34]	0.27
KTR	19	15.49 [9.33; 21.92]	60	5.85 [1.89; 12.27]	**< 0.001**
HD	23	19.01 [12.97; 23.35]	76	14.97 [6.75; 19.88]	**0.009**
Control	23	15.13 [7.11; 20.50]	112	20.57 [16.07; 22.16]	**0.004**
**IgG antibodies against NP**					
Overall	66	35.10 [12.97; 38.27]		NA	
KTR	20	20.70 [8.30; 36.78]		NA	
HD	23	35.81 [33.15; 39.11]		NA	
Control	23	34.51 [15.10; 37.87]		NA	

Table showing the titres (median values, signal/noise ratio) of IgG antibodies directed to RBD, S1S2 and NP of SARS-CoV-2 in responders. Time from PCR or first vaccine to blood sample was not correlated with the IgG RBD titres (*n* = 248, r_s_ = 0.04, *p* = 0.50) while it was weakly correlated with the IgG S1S2 titres (*n* = 236, r_s_ = 0.17, *p* = 0.01). *KTR* Kidney transplant recipients, *HD* hemodialysis

**Table 3:** Proportion of patients developing IgA antibodies against SARS-CoV-2 after infection and vaccination

	Infected		Vaccinated		
	*N*	*n* (%)	*n*	*n* (%)	*p*-value
**IgA antibody seroconversion rate against RBD**					
Overall	68	52 (76.5)	247	102 (41.3)	**< 0.001**
KTR	21	17 (80.9)	129	57 (44.2)	**0.002**
HD	24	18 (75.0)	85	38 (44.7)	**0.009**
Control	23	17 (73.9)	33	7 (21.2)	**< 0.001**
**IgA antibody seroconversion rate against S1S2**					
Overall	68	60 (88.4)	247	122 (49.4)	**< 0.001**
KTR	21	19 (90.5)	129	57 (44.2)	**< 0.001**
HD	24	21 (87.5)	85	50 (58.8)	**0.009**
Control	23	20 (87.0)	33	15 (45.5)	**0.002**

Table showing seroconversion IgA antibodies directed to RBD and S1S2 of SARS-CoV-2. *KTR *Kidney transplant recipients, *HD* hemodialysis

**Table 4:** IgA antibody levels against SARS-CoV-2 in responders after infection and vaccination

	Infected			Vaccinated			
	*N*		Median [IQR]	*n*		Median [IQR]	*p*-value
**IgA antibody titres against RBD**							
Overall	52	2.97 [1.55; 7.37]		102	2.91 [1.61; 4.94]		0.39
KTR	17	7.09 [2.28; 10.09]		57	3.11 [1.75; 4.70]		**0.009**
HD	18	2.19 [1.45; 6.75]		38	2.99 [1.87; 6.64]		0.54
Control	17	2.64 [1.60; 5.08]		7	1.46 [1.20; 1.54]		**0.01**
**IgA antibody titres against S1S2**							
Overall	60	5.82 [2.75; 14.10]		122	2.29 [1.41; 4.15]		** < 0.001**
KTR	19	12.41 [2.78; 21.10]		57	2.43 [1.57; 3.42]		** < 0.001**
HD	21	4.66 [2.54; 11.84]		50	2.29 [1.38; 4.32]		**0.01**
Control	20	4.41 [2.80; 8.05]		15	1.61 [1.24; 4.15]		**0.004**

Table showing the IgA antibody titres directed to RBD (upper panel) and S1S2 (lower panel) of SARS-CoV-2 in responders. Time from PCR or first vaccine to blood sample was not correlated with the IgA RBD titres (*n* = 154, r_s_ = 0.02, *p* = 0.82) nor IgA S1S2 titres (*n* = 182, r_s_ = -0.08, *p* = 0.27). *KTR* Kidney transplant recipients, *HD* hemodialysis

Within the SECRET study, due to logistic reasons, it was not always possible to sample patients according to the protocol and therefore, there was some variation in time between COVID-19 diagnosis and sampling time. We therefore analyzed the relation between timepoint of infection or first vaccine to determination of antibody titres. The time after either a positive PCR test or first vaccine to blood sample was not related to the IgA anti-RBD or anti S1S2 titres nor the IgG anti-RBD titres. A weak correlation between time to blood sample and IgG anti-S1S2 Ab titres was found (*n* = 236, r_s_ = 0.17, *p* = 0.01). We therefore didn’t compare the IgG S1S2 antibody titres over time between subgroups. The time difference did not influence the neutralizing antibody capacity. Since blood samples were collected according to the predefined protocol for the vaccination studies, in the next part, we only report the titres within each subgroup of the UPRAISE and COVEMUZ study.

**Fig. 1:**
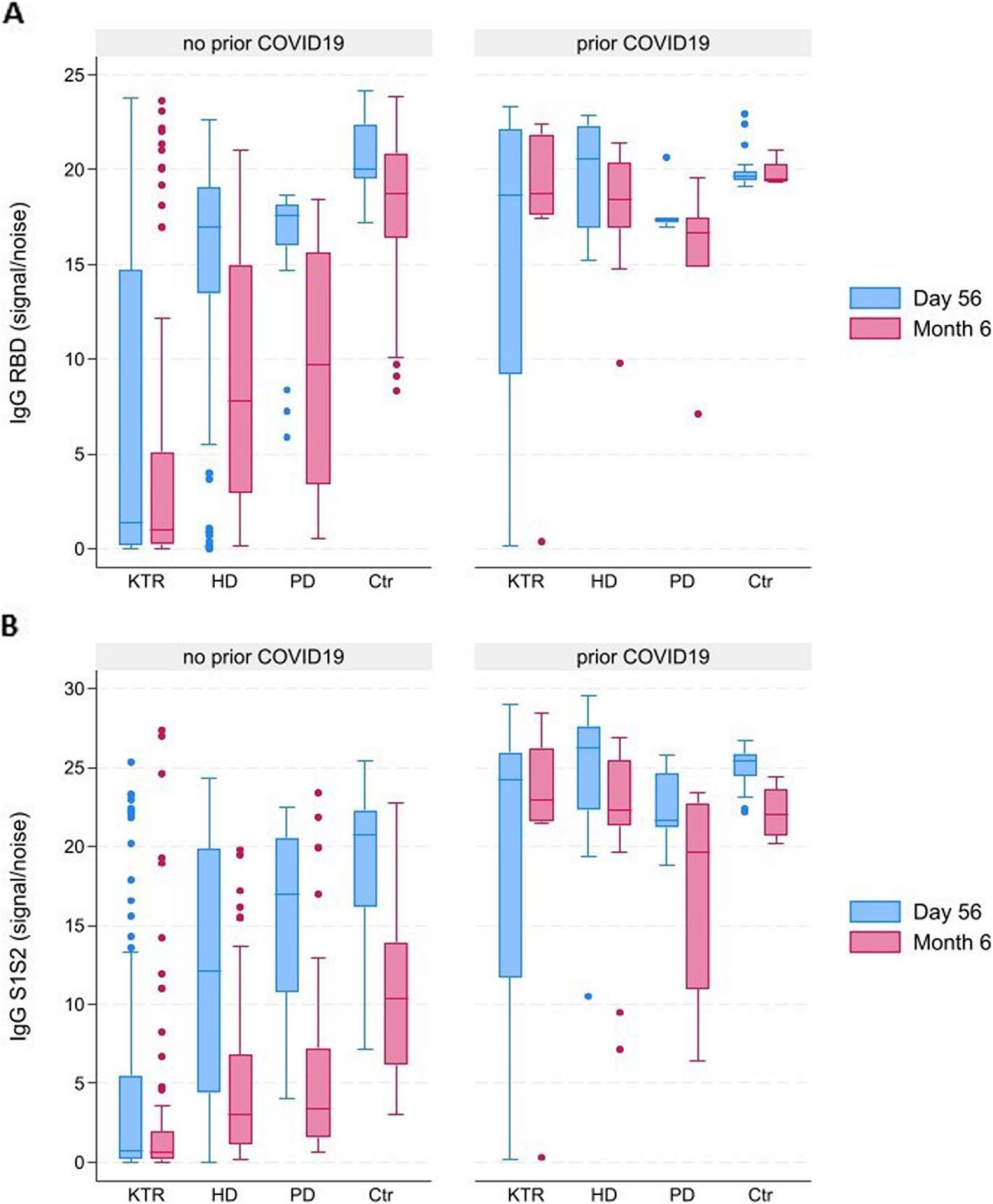
Graph showing the evolution of titres of IgG antibodies directed to RBD (**A**) and S1S2 (**B**). Blood sampling and measurements were done at 56 days (blue bars) and six months (red bars) after two-dose mRNA vaccination. KTR: kidney transplantation recipient; HD: hemodialysis; PD: peritoneal dialysis; Ctr: controls, NT50: neutralizing capacity

2. Immune response over a six months time period after a 2-dose SARS-CoV-2 mRNA vaccination.
  - Patients

- In this part of the study, patients from UPRAISE and COVEMUZ were included. In total, 506 patients were included of whom 28 were excluded for the following reasons: 26 patients on dialysis with a history of kidney transplantation, two patients who switched groups during the course of the study. 478 patients were included in the analysis (153 kidney transplant recipients, 115 patients on haemodialysis, 37 patients on peritoneal dialysis and 173 controls). Baseline characteristics of the study population are shown in Supplemental Table 4.
  b. Humoral antibody titres over time.

- The evolution of (neutralizing) antibody responses after vaccination in all subgroups was studied in patients without and with prior evidence of exposure to SARS-CoV-2. A PCR-proven history of SARS-CoV-2 infection or positivity for anti-RBD IgG antibodies (> 1) at the day of vaccination without a PCR-proven history of SARS-CoV-2 infection, were defined as prior COVID-19 infection.
- In individuals without prior COVID-19, the anti-RBD and anti-S1S2 IgG were significantly lower after vaccination in KTR when compared to any other group at each time point (p_adj_ < 0.001). In individuals with prior COVID-19, IgG anti-RBD, IgG anti-S1S2 (Fig. 1 A and B) and neutralizing capacity (Supplemental Fig. 1) did not differ between groups (*p* > 0.05).
- Both in individuals without or with prior COVID-19, the IgG anti-RBD and S1S2 antibody levels significantly decreased over time (Fig. 1 A and B; in individuals without prior COVID-19 for both outcomes: p < 0.001 for HD, PD and Ctr and p = 0.002 for KTR). In individuals without prior COVID-19, neutralizing capacity (Supplemental Fig. 1) declined after six months in both dialysis groups and the control group (*p* < 0.001 for HD, *p* = 0.005 for PD, *p* < 0.001 for control), and remained stable in the KTR group (*p* = 0.15). Descriptive statistics and *p*-values for the comparisons according to prior COVID-19 are provided in Supplemental Table 5.
- In individuals with a humoral antibody response but without prior COVID-19, neutralizing capacity was present in 91.9% of vaccinated individuals after 56 days and in 63.4% after six months. In individuals with a humoral antibody response and evidence of prior COVID-19 disease, neutralizing responses were present in 100% after 56 days and 86.7% of individuals after six months.
  c. Variables associated with SARS-CoV-2 antibody responses after vaccination

**Table 5:** Multivariable analysis of the titre level of SARS-CoV-2 IgG anti-RBD in responders

Variable	Anti-RBD IgG titre b [95% CI]	*p*-value
Patient type		< 0.001*
KTR	-8.15 [-9.43; -6.88]	
HD	-3.04 [-4.37; -1.73]	
PD	-3.48 [-5.27; -1.68]	
Ctr	0	
Age	-0.04 [-0.08; -0.00]	**0.001**
Evidence of prior COVID-19 exposure	2.22 [0.97; 3.46]	** < 0.001**

Table showing multivariable model with IgG anti-RBD as the outcome variable in responders. KTR: kidney transplant recipient; *HD* patient on hemodialysis, *PD* patient on peritoneal dialysis, *Ctr* control. *p_adj_ all < 0.001 except PD vs Ctr 0.001 and HD vs PD 1.00 (*n* = 369)

We investigated which variables are related to antibody dynamics after vaccination. In a first analysis, the presence of anti-RBD IgG was analyzed as an outcome variable in 456 patients (data shown in Supplemental Table 6). Evidence of prior COVID-19 was associated with the presence of anti-RBD IgG (OR = 7.82 [95% CI 1.87; 32.69], *p* = 0.005). In comparison to the intake of CNI and/or corticosteroids, the additional intake of MMF (mycophenolate mofetil) or AZA (azathioprine) was associated with significantly lower chance of developing IgG RBD (OR = 0.12 [95% CI 0.04; 0.31], *p* < 0.001). Also, being a kidney transplant recipient was associated with a lower chance (59.6%) to develop anti-RBD IgG when compared to patients on haemodialysis (92.2%) or patients on peritoneal dialysis (100%) or healthy controls (100%) (all p_adj_ < 0.001). Being on haemodialysis was associated with a lower chance to develop anti-RBD IgG when compared to healthy controls (p_adj_ < 0.001) (no odds ratio according to patient type was calculable because all patients on peritoneal dialysis and healthy controls developed anti-RBD IgG).

Next, we investigated which variables were predictive for levels of anti-RBD IgG titres (among patients with a positive antibody response; *n* = 386). The following variables were significantly associated with anti-RBD IgG titres (Supplemental Table 7): type of patient (with KTR having the lowest Ab titres and controls the highest; *p* < 0.001), prior COVID-19 (higher mean Ab titre; b = 2.63 [95% CI 1.14; 4.11], *p* = 0.001), type of vaccine (lower antibody titres after vaccination with mRNA-1273; b = -2.19 [95% CI -3.50; -0.89], *p* = 0.001) and age (with older individuals having lower Ab titres; b = -0.05 [95% CI -0.09; -0.01], *p* = 0.02). In the multivariable model (Table 5), vaccine type was left out as it was no longer associated with the IgG anti-RBD antibody level. As shown in Supplemental Table 8, the same variables were related to anti-S1S2 IgG levels.

In a last analysis (Supplemental Table 9), the associations of variables with the level of neutralizing antibody capacity were analyzed within the subgroup of patients who had a positive neutralization test (neutralizing capacity greater than 49) after the first vaccine (*n* = 117, see Supplemental Table 9). Evidence of prior COVID-19, having T2D and higher levels of anti-S1S2 IgG were associated with the neutralizing capacity in the separate analyses, however only the IgG anti-S1S2 level at day 56 after the first vaccine (b = 43.47 [95% CI 30.14; 56.81], *p* < 0.001) and prior COVID-19 (b = 832.25 [95% CI 685.84; 978.66] *p* < 0.001) were associated with neutralizing antibody capacity after adjustment for the other variables in a multivariable model.

## Discussion

This large prospective multicentre study in patients on renal replacement therapy (kidney transplantation, haemodialysis or peritoneal dialysis) shows that the proportion of patients who developed IgG RBD and IgG S1S2 antibodies titres against SARS-CoV-2 were higher after infection (86.4%) than after a two-dose vaccination (55.8%) in kidney transplant recipients (KTR), while seroconversion was comparable in patients on haemodialysis (HD) after infection (95.8%) versus vaccination (89.4%). Healthy controls had a 100% seroconversion rate in both conditions. So indeed, the immune response was overall weaker in our immunodeficient population with a better immune response in HD compared to KTR after a two-dose vaccination. In individuals without prior COVID-19, the anti-RBD and anti-S1S2 IgG level was significantly lower in KTR when compared to all other groups. In patients with a proven COVID-19 infection prior to vaccination, the antibody levels were higher in all patient groups from first measurement point on and remained high at six months after vaccination. Another study confirms higher anti-Spike IgG levels in KTR with prior COVID-19 after a single dose of a SARS-CoV-2 mRNA-based vaccine [27]. Our study and previous reports confirm that hybrid immunity is the best possible defence against severe COVID-19 disease and seems also to hold up for this population. Alternatively, the administration of (preventive) monoclonal antibodies against SARS-CoV-2 has been proven to be effective in immunocompromised populations, [11] however, as this strategy is beyond the scope of the present study, we will not go into further detail.

This study showed that more KTR developed anti-RBD and anti-S1S2 IgG after SARS-CoV-2 infection than after a two-dose vaccination. In addition, these antibody levels were significantly lower after vaccination compared to infection. Both results indicate that the immune response is triggered more efficiently during natural infection in KTR, during which the virus immediately encounters the Nose- and Bronchus-Associated Lymphoid Tissue (NALT and BALT) and triggers an unspecific IgM response. As the B cells mature, they differentiate into plasma cells and produce IgG and IgA antibodies directed to different viral antigens such as Spike (which includes S1S2 and RBD) and NP [19, 28]. Although the natural immunological response seems more efficient than after vaccination in KTR, infection should be avoided in this vulnerable population since immunosuppression carries a greater risk of complications attributable to SARS-CoV-2 infection [6, 7] with mortality rates around 25% [8–10]. Next to KTR, the anti-S1S2 IgG levels of HD patients were significantly higher after infection while the anti-RBD IgG level was comparable after infection vs. vaccination. In both patient groups, post-infection antibody dynamics seemed to have a slower decline than post-vaccination titres [29, 30]. It has been proposed that natural infection provides broader and longer-lasting immune responses in both KTR and HD patients, protecting against hospital admission or severe disease compared to vaccination alone. Nevertheless, it should be taken into account that the initial antibody levels provided by an infection are heavily dependent upon the severity of the initial infection, implying that asymptomatic or mild infections may not provide robust protection [31]. Interestingly, the IgG levels of healthy controls along with their neutralizing capacity were higher after vaccination than after infection. Probably, the immunocompromised state of KTR and HD patients might significantly lower the development of an effective humoral immune response after vaccination, compared to natural infection. This assumption corresponds with the findings of a recent vaccination study, which looked at the IgG and cellular responses after a double-dose mRNA vaccination [17]. Therefore, an effective vaccination strategy is important in these populations. The larger Spanish multicenter Sencovac study recruited 1746 patients with end-stage renal disease among 50 different Spanish centers to examine IgG antibodies to SARS-CoV-2 Spike protein after four vaccine doses and showed elevated antibody titers, especially in HD patients but not in KTR and PD patients, after the fourth dose. Seroconversion was achieved in 72% of previously negative patients after the fourth dose [32].

In all study subgroups, both IgA seroconversion rate and titres were higher after infection. The mucosa is directly targeted during natural infection, leading to the release of secretory IgA which plays a major role in the protection of mucosae against pathogens. The IgA titres were the highest in KTR, both after infection and vaccination. In a previous report, KTR did show early IgA responses after COVID-19, whereas IgG responses were delayed [33]. Besides, IgA levels in vaccinated solid organ transplants (SOT) [34] and health care workers [35] were higher in case of a prior SARS-CoV-2 infection. Cravedi et al*.* stated that IgA responses can occur independently of T-cell mediated pathways which might be the reason why this Ig class is less affected by immunosuppressive therapy [33]. On the one hand, it has been shown that IgA has more potential in neutralizing activity against SARS-CoV-2 [21] but on the other hand, it has been shown that S1-IgA levels are predictive for the clinical manifestations of COVID-19 with higher levels being associated with a worse course of the disease [36]. It is thus not clear if the higher IgA levels in our KTR subgroup is beneficial for neutralizing SARS-CoV-2 or if it is a sign of disease severity. For instance, it has been postulated that enhanced IgA antibody formation after COVID-19 might contribute to Immunoglobulin A nephropathy (IgAN), newly induced or as an exacerbation of a pre-existing IgAN [37]. Since we did not take the course of the disease into account in the analyses performed in this study, we cannot elaborate on that.

In the second part of this study, we investigated the effect of a prior COVID-19 on the antibody responses generated by two-dose mRNA vaccination over a 6-months’ follow-up period in a large cohort of KTR, HD, PD and controls. People with prior exposure to COVID-19 had overall higher and more stable IgG titres than individuals without prior exposure, along with an enhanced neutralizing capacity. Of note, the neutralization antibody analyses were performed in a rather small subgroup of subjects with a positive humoral response and need to be interpreted in that context. Furthermore, we found that the anti-RBD and anti-S1S2 IgG were significantly lower after vaccination in KTR when compared to any other group at each time point. This has also been noted by a recent nine-month observational study [38], however they also found that the HD group was at specific risk for strong decline of RBD antibodies. Therefore, especially the HD patients might benefit from a third vaccine dose to increase the RBD antibody titers, along with naïve COVID-19 patients to enhance the IgG neutralizing capacity and protection against reinfection [39]. Six months after the double-dose vaccination, the neutralizing capacity in HD patients without vs with prior exposure was no longer significant, a phenomenon that was not seen for the IgG titres. It thus seems that the development of immunological memory does not impact the neutralizing antibody capacity as much as the IgG titers. A two-dose intramuscular mRNA vaccination was performed, however, a recent pediatric study showed that an alternative administration route via intradermal SARS-CoV-2 vaccination could be beneficial compared to intramuscular vaccination with superior antibody responses [40]. Nevertheless, this proposed method requires further research [41].

Furthermore, it is important to keep in mind that many variables can affect antibody titres after vaccination. Multiple determinants including nonmodifiable (e.g. sex, age) and clinical (e.g. patient type, comorbidities, vaccine type) factors may play a key role in the kinetics of the humoral response and may determine the basis of a heterogeneous antibody production [29, 31]. In this study, we showed that kidney transplantation was negatively associated with IgG titres, followed by peritoneal dialysis and then haemodialysis. Prior SARS-CoV-2 infection was positively associated with antibody titres. Increasing age negatively influenced the IgG titres, which can be explained by the gradual development of ‘immunosenescence’ [42]. This age-related phenomenon primarily causes problematic cellular immunity, but results in less efficient T-cell dependent B cell responses and antibody responses as well [42–44]. Besides kidney transplantation or dialysis and age, also the mRNA-1273 (Moderna) vaccine was negatively associated with IgG titres when compared to the BNT162b2 (Pfizer/BioNTech) vaccine. Previous studies of our own research group and others showed that the seroconversion rate in SOT was better after mRNA-1273 than after BNT162b2 vaccination [12, 45]. However, in the multivariable analysis, after correction for the other variables in the model, this association was lost. Finally, we found that the seroconversion rate after vaccination was negatively associated with the intake of AZA (azathioprine) or MMF (mycophenolate mofetil) additionally to CNI and/or corticosteriods, while in responding patients the antibody titres were not influenced by these drugs. The latter is supported by a recent RCT, in which it was shown that pausing AZA or MMF for two weeks in KTR to three or four doses of a SARS-CoV-2 vaccine did not influence response to a subsequent vaccination [46].

This study assembled a unique and large population of kidney transplant recipients, haemodialysis and peritoneal dialysis patients and controls. Samples were collected prospectively immediately after the appearance of the first cases of COVID-19 in our vulnerable population of renal replacement therapy patients, allowing us to collect data on immunity acquired by infection and after the initial two-dose mRNA vaccination regimen. Our findings make an important contribution to the COVID-19 research field by analysing both IgG and IgA serology and neutralizing antibody capacity after infection and vaccination, leading to a better understanding of the quality of the humoral immune response and its relationship with clinical data. However, this study has some limitations as well. Because the exact date of SARS-CoV-2 infection was not always known, we cannot draw precise conclusions about time-related effects on the antibody response. According to the predetermined protocol, we aimed at sampling patients until one year after infection, however, due to the introduction of the (necessary) vaccination schedule, we could not adhere to the protocol and thus follow-up samples were missing in the SECRET study. The same was true for the UPRAISE study, where we planned to sample patients for one year after the two-dose schedule, but due to the early introduction of the third dose in our patient subgroups with immunodeficiency, we had to stop the study six months earlier. Next, we observed considerable missing data, mainly among the neutralization responses six months after double-dose vaccination resulting in a small number of observations for some analyses, which may result in a lack of significance due to low power. Another limitation is that patients who were not able to produce antibodies could still have a detectable vaccine-specific T cell response, which might be sufficient to prevent severe COVID-19 [47]. T cell responses to SARS-CoV-2 were not investigated in our article.

In conclusion, this prospective multicenter study in renal replacement therapy patients reveals that the humoral immune response to SARS-CoV-2 in kidney transplant recipients was more robust following infection than vaccination, while patients on haemodialysis exhibited comparable seroconversion rates. Notably, individuals with prior COVID-19 exhibited higher anti-RBD and anti-S1S2 IgG levels. Hybrid immunity is thus the best possible defence against severe COVID-19 disease and seems also to hold up for these populations. Interestingly, the IgA titres were the highest in KTR compared to any other group at each time point, both after infection and vaccination, while the IgG titres after vaccination were the lowest. This corresponds with previous findings, in which KTR showed early IgA responses after COVID-19, whereas IgG responses were delayed [33]. However, it is not clear whether the higher IgA levels is beneficial for neutralizing SARS-CoV-2 or if it is a sign of disease severity. To be able to make a statement about the correlation between IgG and IgA antibodies and the course of COVID-19 diseases, it would have been of value to analyze the incidence and severity of further COVID infections. These parameters were not included in the design of our study. Nevertheless, future research should explore long-term immune responses in these patients and the effects of additional vaccine doses, considering individual factors including age and immunosuppressive regimens, in order to design more effective strategies for protection against reinfection. Finally, our results strengthen the need to administer the recommended vaccines to patients with end-stage renal disease (ESRD), and to consider booster vaccination [17, 38, 48].

### Supplementary Information

Supplementary Material 1.

## Data Availability

The data underlying this article will be shared on reasonable request to the corresponding author.
