# Supplementary material for: Humoral immunity to SARS-CoV-2 in kidney transplant recipients and dialysis patients: IgA and IgG patterns unraveled after SARS-CoV-2 infection and vaccination
Source: Virol J. 2024 Jun 13;21:138. doi: 10.1186/s12985-024-02410-1 (PMC11170792; doi:10.1186/s12985-024-02410-1)
Supplement: Supplementary file 1 — Supplementary Material 1. [file 12985_2024_2410_MOESM1_ESM.docx]

**Supplementary tables**

|  |  | Infected (n = 69) | | | Vaccinated (n = 326) | | |
| --- | --- | --- | --- | --- | --- | --- | --- |
|  |  | KTR (n = 22) | HD (n = 24) | Controls (n = 23) | KTR (n = 129) | HD (n = 85) | Controls (n = 112) |
| Female (n (%)) |  | 6 (27.3) | 10 (41.6) | 9 (39.1) | 54 (41.9) | 33 (38.8) | 85 (78.9) |
| Age (y; median [IQR]) |  | 53.5 [38-62] | 68.5 [53-76.5] | 54 [36-61] | 57 [48-65] | 71 [62-77] | 55 [50-61] |
| Comorbidities (n (%)) | T1D | 1 (4.5) | 2/4 (50) | NA | 6 (4.7) | 7/78 (9) | NA |
|  | T2D | 3 (13.6) | 0/4 (0) | NA | 7/129 (5.4) | 31/78 (39.7) | NA |
|  | AID | 1 (4.5) | 1/4 (25) | NA | 10 (7.8) | 6/78 (7.7) | NA |
|  | Other | 21 (95.5) | 3/4 (75) | NA | 125 (96.9) | 77/78 (98.7) | NA |
| Years since last KTR (y; median [IQR]) |  | (n = 4)  6.52 [3.80-10] | NA | NA | 6.87 [3.26-14.09] | NA | NA |
| Total n° of KTx (n (%)) | 1 | 17 (77.3) | NA | NA | 115 (89.1) | NA | NA |
|  | 2 | 3 (13.6) | NA | NA | 8 (6.2) | NA | NA |
|  | 3 | 2 (9.1) | NA | NA | 6 (4.7) | NA | NA |
| Years on dialysis (y; median [IQR]) |  | NA | (n = 4)  3.14 [1.35-4.48] | NA | NA | (n = 84)  2.86 [1.30-5.16] | NA |
| Vaccine-type (n (%)) | BNT162b2 | NA | NA | NA | 89 (69) | 53 (62.3) | 110 (98.2) |
|  | mRNA-1273 | NA | NA | NA | 40 (31) | 32 (37.7) | 2 (1.8) |
| Immunosuppressive therapy (n (%)) | Oral CS and/or CNI | 7 (31.8) | NA | NA | 39/128 (30.5) | 1 (1.2) | NA |
|  | Oral CS/CNI + MMF or AZA | 15 (68.2) | NA | NA | 89/128 (69.5) | 4 (4.7) | NA |

**Supplemental table 1:** Baseline characteristics of the patients included in the analysis of humoral immune response after vaccinations versus after infection. KTR : kidney transplant recipient ; HD : hemodialysis ; T1D: type 1 diabetes; T2D: type 2 diabetes ; KTx: kidney transplantation; CNI: calcineurin inhibitor; CS: corticosteroids; MMF: mycophenolate mofetil; AZA: azathioprine; AID: autoimmune disease

| Infected | | | Vaccinated | | |  |
| --- | --- | --- | --- | --- | --- | --- |
| n | r_s_ | p | n | r_s_ | p |  |
| **Correlation between neutralizing capacity and IgG anti-RBD titres** | | | | | | |
| Overall | 61 | 0.49 | **< 0.001** | 145 | 0.10 | 0.23 |
| KTR  HD  Control | 19  23  19 | 0.11  0.64  0.72 | 0.67  **< 0.001**  **< 0.001** | 17  21  107 | 0.08  -0.33  0.22 | 0.77  0.14  **0.02** |
| **Correlation between neutralizing capacity and IgG anti-S1S2 titres** | | | | | | |
| Overall | 61 | 0.37 | **0.003** | 145 | 0.67 | **< 0.001** |
| KTR  HD  Control | 19  23  19 | 0.22  0.47  0.51 | 0.37  **0.03**  **0.03** | 17  21  107 | 0.78  0.41  0.67 | **< 0.001**  0.07  **< 0.001** |

**Supplemental table 2:** Correlation between neutralizing capacity and IgG antibody levels against SARS-CoV-2 in responders after infection and vaccination. Table showing the correlation coefficients between IgG anti-RBD (upper panel) and IgG anti-S1S2 titres (lower panel) and the neutralizing antibody capacity (NT50) in responders. Time from PCR or first vaccine to blood sample was not correlated with the neutralizing capacity (n = 129, r_s_ = -0.03, p = 0.74) nor IgG anti-RBD titres (n = 129, r_s_ = 0.006, p = 0.74) nor IgG anti-S1S2 titres (n = 129, r_s_ = 0.12, p = 0.17). r_s_: Spearman correlation coefficient; KTR : Kidney transplant recipients; HD: hemodialysis; NT50: neutralizing capacity against SARS-CoV-2

| **A** | Neutralizing capacity  Percent diff [95% CI] | p-value |
| --- | --- | --- |
| anti-RBD IgG titre  Infected or vaccinated  Infected  Vaccinated  Patient type  KTR  HD  Ctr | 7.17 [3.89 ; 10.55]  0  -36.16 [-52.02 ; -15.06]  18.15 [-12.98 ; 60.41]  14.12 [-14.54 ; 52.38]  0 | **< 0.001**  **0.002**  0.47 |
| **B** | Neutralizing capacity  Percent diff [95% CI] | p-value |
| anti-S1S2 IgG titre  Infected or vaccinated  Infected  Vaccinated  Patient type  KTR  HD  Ctr | 8.98 [6.9 ; 11.09]  0  -31.67 [-45.81 ; -13.83]  27.20 [-3.16 ; 67.06]  -8.44 [-28.97 ; 18.03]  0 | **0.001**  **< 0.001**  0.10 |

**Supplemental table 3:** multiple linear regression of the neutralizing capacity including all patients (IgG responders with neutralizing capacity, n=206). To normalize the residuals, we analyzed the natural logarithm of the neutralizing capacity. We back-transformed the regression coefficients in order to report the percentage difference in neutralizing capacity (in terms of geometric means). Anti-RBD IgG titre and anti-S1S2 IgG titre were correlated (Spearman’s rho in Ig G responders = 0.68, p < 0.001), we therefore present two separate multivariable models with either one (panel A for anti-RBD IgG and panel B for antiS1S2 IgG titres). KTR : Kidney transplant recipients; HD: hemodialysis

| Variables | KTR (n = 153) | PD (n = 37) | HD (n = 115) | Controls (n = 173) |
| --- | --- | --- | --- | --- |
| Female (n (%)) | 65 (42.5) | 12 (32.4) | 43 (37.4) | 128 (74.0) |
| Age (y; median [IQR]) | 57 [47 ; 64] | 71 [60 ; 76] | 71 [62 ; 79] | 55 [50 ; 61] |
| Comorbidities (n (%))  T1D  T2D  AID  Other | 7 (4.6)  12 (7.8)  11 (7.2)  147 (96.1) | 3 (8.1)  11 (29.7)  5 (13.5)  35 (94.6) | 9/100 (9.0)  41/100 (41)  8/100 (8)  98/100 (98) | NA |
| Years since last KTx (y; median [IQR]) | 6.34 [3.09 ; 12.81] | NA | NA | NA |
| Total no. of KTx (n (%))  1  2  3 | 136 (88.9)  10 (6.5)  7 (4.6) | NA | NA | NA |
| Years on dialysis (median [IQR]) | NA | 1.71 [1.09 ; 2.83] | 2.70 [1.37 ; 5.16] | NA |
| Evidence of prior COVID-19 (n (%)) | 12/146 (8.2) | 5/35 (14.3) | 27/114 (23.7) | 27/146 (18.5) |
| Vaccine-Type (n (%))  BNT162b2  mRNA-1273 | 104 (68.0)  49 (32.0) | 17 (45.9)  20 (54.1) | 71 (61.7)  44 (38.3) | 171 (98.8)  2 (1.2) |
| Ab response to vaccine  (RBD positivity) (n (%)) | 73/132 (55.3) | 30/30 (100.0) | 78/87 (90.0) | 119/119 (100) |
| Immunosuppressive therapy (n (%))  Oral CS and/or CNI  Oral CS/CNI + MMF or AZA | 47/152 (30.9)  105/152 (69.1) | NA | 2 (1.7)  4 (3.5) | NA |

**Supplemental table 4:** Baseline characteristics of the patients included in the analysis of the evolution of humoral immune response after vaccination. KTR : kidney transplant recipient ; HD : hemodialysis ; T1D: type 1 diabetes; T2D: type 2 diabetes ; KTx: kidney transplantation; CNI: calcineurin inhibitor; CS: corticosteroids; MMF: mycophenolate mofetil; AZA: azathioprine; AID: autoimmune disease

| Without prior COVID-19 exposure | | With prior COVID-19 exposure | |  |  |
| --- | --- | --- | --- | --- | --- |
| N | Median [IQR] | N | Median [IQR] | p-value |  |
| **IgG anti-RBD antibody titres in subjects with and without prior exposure to COVID-19** | | | | | |
| **IgG anti-RBD titres day 56** | | | | | |
| Overall | 368 | 17.56 [3.94 ; 19.92] | 71 | 19.66 [17.47 ; 21.08] | **< 0.001** |
| KTR  HD  PD  Control | 132  87  30  119 | - 1. [0.22 ; 14.70]   16.98 [13.46 ; 19.06]  17.56 [16.01 ; 18.14]  20.05 [19.57 ; 22.39] | 12  27  5  27 | 18.69 [9.23 ; 22.14]  20.55 [16.96 ; 22.30]  17.33 [17.21 ; 17.47]  19.67 [19.43 ; 19.90] | **0.002**  **< 0.001**  0.78  **0.02** |
| **IgG anti-RBD titres month six** | | | | | |
| Overall | 240 | 6.15 [0.91 ; 16.42] | 35 | 19.05 [16.93 ; 20.38] | **< 0.001** |
| KTR  HD  PD  Control | 96  77  28  39 | 0.99 [0.23 ; 5.09]  7.79 [2.94 ; 14.97]  9.73 [3.42 ; 15.61]  18.71 [16.43 ; 20.87] | 8  18  5  4 | 18.77 [17.62 ; 21.83]  18.45 [16.93 ; 20.38]  16.64 [14.90 ; 17.46]  19.46 [19.38 ; 20.28] | **< 0.001**  **< 0.001**  0.09  0.18 |
| **IgG anti-S1S2 antibody titres in subjects with and without prior exposure to COVID-19** | | | | | |
| **IgG anti-S1S2 titres day 56** | | | | | |
| Overall | 368 | 13.47 [1.55 ; 20.60] | 71 | 25.23 [22.73 ; 26.46] | **< 0.001** |
| KTR  HD  PD  Control | 132  87  30  119 | 0.73 [0.23 ; 5.49]  12.15 [4.40 ; 19.83]  16.98 [10.82 ; 20.55]  20.73 [16.15 ; 22.30] | 12  27  5  27 | 24.19 [11.72 25.93]  26.21 [22.32 ; 27.62]  21.63 [21.20 ; 24.63]  25.44 [24.49 ; 25.87] | **< 0.001**  **< 0.001**  **0.008**  **< 0.001** |
| **IgG anti-S1S2 titres month six** | | | | | |
| Overall | 240 | 2.53 [0.61 ; 7.28] | 35 | 22.21 [21.15 ; 25.00] | **< 0.001** |
| KTR  HD  PD  Control | 96  77  28  39 | 0.63 [0.21 ; 1.94]  3.04 [1.16 ; 6.81]  3.39 [1.58 ; 7.22]  10.38 [6.18 ; 13.91] | 8  18  5  4 | 22.98 [21.58 ; 26.22]  22.29 [21.33 ; 25.43]  19.62 [10.99 ; 22.73]  22.05 [20.71 ; 23.61] | **< 0.001**  **< 0.001**  **0.01**  **0.004** |
| **Neutralization capacity in subjects with and without prior exposure to COVID-19** | | | | | |
| **NT50 day 56** | | | | | |
| Overall | 101 | 213.91 [100.00 ; 340.87] | 18 | 1601 [1224.43 ; 1601] | **< 0.001** |
| KTR  HD  PD  Control | 22  25  24  30 | 125.89 [57.43 ; 269.18]  213.91 [93.30 ; 297.20]  175.27 [83.23 ; 377.99]  288.59 [150.54 ; 400.00] | 7  5  3  3 | 1601 [1600 ; 1601]  1601 [1601 ; 1601]  459.48 [340.87 ; 666.61]  1601 [1062.83 ; 1601] | **< 0.001**  **< 0.001**  0.12  **0.005** |
| **NT50 month six** | | | | | |
| Overall | 71 | 70.71 [49 ; 137.96] | 15 | 800 [200 ; 1369.77] | **< 0.001** |
| KTR  HD  PD  Control | 9  20  17  25 | 112.08 [49.00 ; 150.54]  62.05 [49.00 ; 74.63]  49.00 [49.00 ; 163.54]  100.00 [61.11 ; 125.56] | 5  4  3  3 | 1233.77 [800 ; 1392.88]  670.31 [156.45 ; 1223.25]  200 [66.43 ; 261.35]  1204.33 [400 ; 1601] | **0.04**  0.06  0.24  **0.005** |

**Supplemental table 5:** Table showing the titres (median values, signal/noise ratio) of IgG antibodies directed to RBD, NP and S1S2 of SARS-CoV-2 and neutralizing capacity on day 56 and month six . KTR: Kidney transplant recipients; HD: hemodialysis; PD: peritoneal dialysis

| Variable | n | Presence of RBD IgG  OR [95% CI] | p-value |
| --- | --- | --- | --- |
| Female | 456 | 1.26 [0.76 ; 2.11] | 0.37 |
| Age | 456 | 1.01 [0.99 ; 1.03] | 0.23 |
| Comorbidities  T1D  T2D  AID  Other | 338  338  288  288 | 1.30 [0.37 ; 4.62]  1.71 [0.80 ; 3.67]  0.94 [0.36 ; 2.47]  1.38 [0.35 ; 5.74] | 0.69  0.17  0.90  0.65 |
| Years since last KTx | 151 | 1.03 [0.99 ; 1.08] | 0.12 |
| Total number of KTx | 151 | 0.96 [0.49 ; 1.90] | 0.91 |
| Years on dialysis | 151 | 0.98 [0.79 ; 1.22] | 0.85 |
| Evidence of prior COVID-19 | 439 | 7.82 [1.87 ; 32.69] | **0.005** |
| Type of vaccine  BNT162b2  mRNA-1273 | 456  341  115 | 1  1.06 [0.59 ; 1.92] | 0.85 |
| Immunosuppressive categories  Oral CS and/or CNI  Oral CS/CNI + MMF or AZA | 158  51  107 | 1  0.12 [0.04 ; 0.31] | **< 0.001** |

**Supplemental table 6:** Analyses of the presence of SARS-CoV-2 IgG anti-RBD according to patient characteristics. Table showing Odds ratio with 95% confidence interval which represents the strength of the association between predictor variable and outcome (IgG RBD signal/noise value on day 56). OR: Odds ratio; CI: confidence interval; T1D: type 1 diabetes; T2D: type 2 diabetes; AID: autoimmune disease; KTx: kidney transplant; CNI: calcineurin inhibitors, MMF: mycophenolate mofetil; AZA: azathioprine

| Variable | n | Anti-RBD IgG titre  b [95% CI] | p-value |
| --- | --- | --- | --- |
| Patient type  KTR  HD  PD  Ctr | 386  90  106  37  153 | -8.12 [-9.39 ; -6.85]  -3.57 [-4.77 ; -2.37]  -4.25 [-5.99 ; -2.50]  0 | **< 0.001*** |
| Female | 386 | 0.74 [-0.41 ; 1.89] | 0.21 |
| Age | 386 | -0.05 [-0.09 ; -0.01] | **0.02** |
| Comorbidities  T1D  T2D  Immunodef  Other | 269  269  219  219 | -2.84 [-6.11 ; 0.43]  -0.64 [-2.52 ; 1.22]  -0.42 [-3.53 ; 2.69]  1.52 [-3.34 ; 6.38] | 0.09  0.50  0.79  0.54 |
| Years since last KTx | 90 | -0.02 [-0.24 ; 0.19] | 0.84 |
| Total no. of KTx | 90 | 1.74 [-1.96 ; 5.43] | 0.35 |
| Years on dialysis | 142 | 0.06 [-0.20 ; 0.32] | 0.67 |
| Evidence of prior COVID-19 | 369 | 2.63 [1.14 ; 4.11] | **0.001** |
| Vaccine type  BNT162b2  mRNA-1273 | 386  288  98 | 0  -2.19 [-3.50 ; -0.89] | **0.001** |
| Immunosuppressive categories  Oral CS and/or CNI  Oral CS/CNI + MMF or AZA | 93  44  49 | 0  -0.43 [-3.83 ; 2.96] | 0.80 |

**Supplemental table 7:** Analyses of the titre level of SARS-CoV-2 IgG anti-RBD in responders according to patient characteristics. Table showing regression coefficient b (mean difference) with 95% confidence interval which represents the influence of the predictor variable on the outcome (IgG anti-RBD signal/noise value on day 56) in responders. CI: confidence interval; KTR: kidney transplant recipient; HD: patient on haemodialysis; PD: patient on peritoneal dialysis; Ctr: control; T1D: type 1 diabetes; T2D: type 2 diabetes; Immunodef: immunodeficient; KTx: kidney transplant; CS: corticosteroids; CNI: calcineurin inhibitors, MMF: mycophenolate mofetil; AZA: azathioprine. *p_adj_ all < 0.001 except HD vs PD 1.00.

| Variable | n | Anti-S1S2 IgG titres  b [95% CI] | p-value |
| --- | --- | --- | --- |
| Patient type  KTR  HD  PD  Ctr | 373  77  103  37  153 | -9.47 [-11.38 ; -7.56]  -3.82 [-5.55 ; -2.09]  -4.12 [-6.62 ; -1.62]  0 | **< 0.001** |
| Female | 373 | 1.04 [-0.54 ; 2.62] | 0.20 |
| Age | 373 | -0.07 [-0.12 ; -0.01] | **0.01** |
| Comorbidities  T1D  T2D  Immunodef  Other | 256  256  206  206 | -2.27 [-6.64 ; 2.10]  -0.14 [-2.57 ; 2.30]  -0.32 [-4.49 ; 3.84]  -4.18 [-10.97 ; 2.61] | 0.31  0.91  0.88  0.23 |
| Years since last KTx | 77 | 0.01 [-0.26 ; 0.27] | 0.96 |
| Total no. of KTx | 77 | 2.18 [-1.98 ; 6.34] | 0.30 |
| Years on dialysis | 142 | -0.29 [-0.72 ; 0.15] | 0.19 |
| Evidence of prior COVID-19 exposure | 356 | 9.77 [7.98; 11.56] | **< 0.001** |
| Vaccine-Type  BNT162b2  mRNA-1273 | 373  279  94 | 0  -1.89 [-3.71 ; -0.08] | **0.04** |
| Immunosuppressive categories  Oral CS + CNI  Oral CS + CNI + MMF or AZA | 80  40  40 | 0  0.72 [-3.32 ; 4.76] | 0.72 |

**Supplemental table 8:** Table showing coefficient b with 95% confidence interval which represents the influence of the predictor variable on the outcome (IgG_S1S2 signal/noise value on day 56). CI: confidence interval; KTR: kidney transplant recipient; HD: patient on haemodialysis; PD: patient on peritoneal dialysis; Ctr: control; Immunodef: immunodeficient; KTx: kidney transplant; CNI: calcineurin inhibitors, MMF: mycophenolate mofetil; AZA: azathioprine

| Variable | n | Neutralizing capacity  b [95% CI] | p-value |
| --- | --- | --- | --- |
| Anti-RBD IgG | 117 | 19.95 [-18.82 ; 58.71] | 0.31 |
| Anti-S1S2 IgG | 117 | 73.93 [57.37 ; 90.50] | **< 0.001** |
| Patient type  KTR  HD  PD  Ctr | 117  28  27  26  36 | 189.62 [-47.06 ; 426.29]  100.32 [-138.81 ; 339.44]  -97.05 [-338.79 ; 144.69]  0 | 0.14 |
| Female | 117 | -65.29 [-241.40 ; 110.81] | 0.46 |
| Age | 117 | -0.79 [-6.47 ; 4.89] | 0.78 |
| Comorbidities  T1D  T2D  AID  Other | 116  116  80  80 | 282.44 [-151.75 ; 716.63]  298.06 [46.49 ; 549.63]  -6.29 [-422.82 ; 410.23]  41.77 [-577.66 ; 661.21] | 0.20  **0.02**  0.98  0.89 |
| Years since last KTx | 28 | -25.75 [-58.83 ; 7.33] | 0.12 |
| Total no. of KTx | 28 | 233.55 [-181.30 ; 648.40] | 0.26 |
| Years on dialysis | 53 | -33.39 [-97.18 ; 30.41] | 0.30 |
| Evidence of prior COVID-19 exposure | 109 | 1050.34 [897.32 ; 1203.36] | **< 0.001** |
| Vaccine-Type  BNT162b2  mRNA-1273 | 117  76  41 | 0  9.18 [-175.63 ; 193.99] | 0.92 |
| Immunosuppressive categories  Oral CS and/or CNI  Oral CS/CNI + MMF or AZA | 28  12  16 | 0  129.79 [-373.82 ; 633.41] | 0.60 |

**Supplemental table 9:** Analyses of the neutralizing capacity of SARS-CoV-2 antibodies if positive neutralization test according to patient characteristics. Table showing regression coefficient b (mean difference) with 95% confidence interval which represents the influence of the predictor variable on the outcome (NT50 on day 56) in patients with a positive neutralization test. CI: confidence interval; KTR: kidney transplant recipient; HD: patient on hemodialysis; PD: patient on peritoneal dialysis; Ctr: control; T1D: type 1 diabetes; T2D: type 2 diabetes; AID: autoimmune disease; KTx: kidney transplant; CS: corticosteroids; CNI: calcineurin inhibitors, MMF: mycophenolate mofetil; AZA: azathioprine.

**Supplementary Figures**


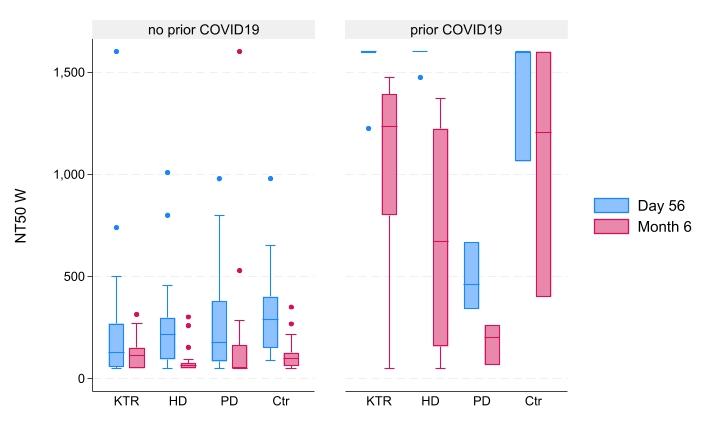


**Supplemental figure 1:** Graph showing the evolution of neutralizing antibody capacity of SARS-CoV-2. Blood sampling and measurements were done at 56 days and six months after two-dose mRNA vaccination. KTR: kidney transplantation recipient; HD: hemodialysis; PD: peritoneal dialysis; Ctr: controls, NT50: neutralizing capacity
